# Identification and Molecular Simulation of Genetic Variants in *ABCA1* Gene Associated with Susceptibility to Dyslipidemia in Type 2 Diabetes

**DOI:** 10.3390/ijms25126796

**Published:** 2024-06-20

**Authors:** Asifa Majeed, Zunaira Ali Baig, Amir Rashid

**Affiliations:** Department of Biochemistry & Molecular Biology, Army Medical College, National University of Medical Sciences, Rawalpindi 46000, Pakistan; zunaira.ali190@gmail.com (Z.A.B.); amir.rashid@numspak.edu.pk (A.R.)

**Keywords:** ApoA1, cholesterol efflux, high-density lipoprotein, LCAT, reverse cholesterol transport

## Abstract

Genetic insights help us to investigate disease pathogenesis and risk. The ABCA1 protein encoded by *ABCA1* is involved in transporting cholesterol across the cell membrane. Genetic variations in the *ABCA1* gene are well documented; however, their role in the development of diabetic dyslipidemia still needs to be explored. This study aimed to identify the associations of rs757194699 (K1587Q) and rs2066714 (I883M) with dyslipidemia in type 2 diabetes and performed molecular simulations. In our case–control study, 330 individuals were divided equally into a diabetic dyslipidemia cases and a healthy controls. Allele-specific polymerase chain reaction and restriction fragment length polymorphism were performed to screen selected variants of the *ABCA1* gene. Sanger sequencing was also performed to find genetic mutations in exon 5 of the *ABCA1* gene. The C allele of rs757194699 was observed at a high frequency in cases compared to controls and followed the overdominant genetic model (*p* < 0.0001, OR:3.84; CI:1.67–8.82). The frequency of G allele of rs2066714 was significantly higher in cases compared to controls and followed the genetic model of codominant (*p*< 0.0001, OR: 39.61; CI:9.97–157.32), dominant (*p* < 0.0001,OR:59.59; CI:15.19–233.81), overdominant (*p*< 0.0001, OR:9.75; CI:3.16–30.11), and log-additive (*p*< 0.0001, OR:42.15; CI:11.08–160.40). In silico modeling and docking revealed that rs2066714 and rs757194699 produced deleterious conformational changes in the ABCA1 protein, resulting in alterations in the binding of the apoA1 protein. There were no genetic variations found in exon-5 in Sanger sequencing. The G allele of rs2066714 and C allele of rs757194699 in the *ABCA1* gene were found to be risk alleles in the development of dyslipidemia in type 2 diabetes. These polymorphisms could alter the binding site of ABCA1 with apoA1 thus disturbs the reverse cholesterol transport.

## 1. Introduction

Diabetes mellitus (DM) is known to be a chronic metabolic syndrome associated with altered glucose metabolism [1]; type 2 has emerged as the most common type [2]. An estimation by the International Diabetes Federation (IDF) predicted over 537 million diabetics in 2021; this figure is projected to increase to 783 million by 2045 [3]. The World Health Organization (WHO) has revealed a dramatic increase in diabetics from 108 million in 1980 to 422 million by the year 2014. There has been an increased mortality rate observed in diabetics, too [4]. It has been classified as an epidemic due to the burden that it has caused on the growing economies of developing countries. A prevalence of 26.3% was reported for diabetes in Pakistan [5]. Various ethnic and environmental factors, such as noise and increased levels of nitrogen dioxide in the air [6] and genetic determinants, are associated to the risk and onset of the disease [7]. Type 2 diabetes mellitus (T2DM) has been expanding globally at an alarming rate and is associated with various micro and macro-vascular complications that have an adverse effect on human health [8]. Dyslipidemia in type 2 diabetes develops as a result of variations in the lipoprotein metabolism and the receptor proteins [9]. The elevated lipid influx in the adipose tissues interrupts normal β-cell function [10]. 

The *ATP-binding cassette transporter A1 (ABCA1)* gene is an important member of the superfamily of *ABC* genes. The gene is located on chromosome 9q31.1. The gene spans the 149 kb region, with 50 exons, and the ABCA1 protein contains 2261 amino acids [11]. The ABCA1 protein, in association with high-density lipoprotein (HDL), takes part in reverse cholesterol transport (RCT) to deliver free cholesterol to the liver for excretion [12,13]. Numerous studies have reported impaired cholesterol efflux due to genetic anomalies in the *ABCA1* gene [14,15]. Genetic and environmental interactions among the *APOA1*, *ABCA1*, and *LCAT* genes have revealed genetic susceptibility to dyslipidemia [16]. Apolipoprotein A-I (apoA1) directly binds to ABCA1 protein located in the plasma membrane and forms HDL [17]. The polymorphisms rs2472386, rs4743763, and rs4149339 in *ABCA1*, in association with confounding factors including physical activity, dessert intake, and fried food intake, were determined to be associated with coronary artery diseases in dyslipidemia patients in southern China [18]. Another study reported specific changes in microRNA and the expression of the *ABCA1* gene in the fetal liver in correlation with maternal obesity and gestational diabetes [19]. In a study, the *ABCA1* gene variant rs2230806 showed a positive association with the levels of plasma triglyceride in patients with severe dyslipidemia, while there was no difference in the genotypic frequencies between cases and controls [20]. The rs2230806 variant of the *ABCA1* gene was also found to be associated with variations in cardio-metabolic traits including obesity, decreased insulin secretion and sensitivity, increased blood glucose levels, and abnormal lipid parameters in gestational diabetic patients [21]. The polymorphisms rs1800977 and rs4149313 in *ABCA1* were found to be positively associated with susceptibility to type 2 diabetes mellitus in subjects in the Chinese Han population [22]. The homozygous TT genotype has been frequently found in the Egyptian population, marking the potential risk that the C69T polymorphism of *ABCA1* among diabetics is associated with dyslipidemia [23]. The non-synonymous *ABCA1* gene variant rs2066714 (I883M), along with rs2230806 and rs2230808, was found to be associated with plasma lipid levels and coronary heart disease in a meta-analysis study [24].

Molecular-profiling-based studies provide a sound insight into the pathogenesis of a particular disorder. Comprehensive molecular approaches pave the way to understanding the role of genetic variants in modulating disease onset. Numerous studies around the world have reported a significantly positive association of *ABCA1* variants with diabetic dyslipidemia. Data on the genetic aspect of diabetic dyslipidemia are greatly lacking for our population. The potential role of genetic predisposition in type 2 diabetics in our local population in the context of abnormal lipid profiles has not been extensively studied. Thus, this study aimed to investigate whether the gene variants rs757194699 (K1587Q) and rs2066714 (I883M) were associated with susceptibility to diabetic dyslipidemia. This study also aimed to identify the genetic variations in exon 5 of the *ABCA1* in type 2 diabetic dyslipidemia patients and molecular simulation of identified variations.

## 2. Results

### 2.1. Genotyping of rs757194699 (K1587Q) of the ABCA1 Gene

There were 87 (79.09%) males and 23 (20.91%) females among the cases and 71 (64.55%) males and 39 (35.45%) females among the control subjects. Table 1 gives the distribution of allelic and genotypic frequencies in the cases and control groups. The C allele was found in 63.3% of the cases compared to 21.3% in the controls. The A allele was found in less count in cases compared to the controls. The CC genotype was found in high frequency in cases (diabetic dyslipidemia) and was absent in the control group. The AC genotype was found in high frequency in cases than controls. The rs757194699 was significantly determined in the dyslipidemia diabetic cases compared to the control and followed the significantly overdominant genetic model (AA-CC/AC, *p* < 0.0001, OR: 3.84; CI:1.67–8.82) (Table 2) adjusted for sex, age, and HDL. There was no significant association found in trend analyses taking sex as a covariate.

### 2.2. Genotyping of rs2066714 (I883M) Variants of the ABCA1 Gene 

The G allele of rs2066714 was significantly observed with the GG (24.5) and AG (70%) genotypes in the cases (diabetic dyslipidemia) compared to the control group. Regarding the G allele, proportion was counted higher in cases than controls as compared to A allele. GG genotype was not detected in controls and only present in cases (Table 1). A significant association of rs2066714 was observed in the codominant (AA/AG/GG, *p* < 0.0001, OR: 39.61; CI:9.97–157.32), dominant (AA/AG-GG, *p* < 0.0001, OR: 59.59; CI:15.19–233.81), overdominant (AA-GG/AG, *p* < 0.0001, OR: 9.75; CI:3.16–30.11), and log-additive (AA = 0, AG = 1, GG = 2, *p* < 0.0001, OR: 42.15; CI:11.08–160.40) genetic models of inheritance (Table 2), and the G allele was observed as a risk allele. The G allele was most frequent in homozygous and heterozygous states in cases compared to controls.

**Table 1:** Allelic and genotypic frequencies of the rs2066714 (N = 220) and rs757194699 (N = 300) variants of the *ABCA1* gene.

SNP	Groups	
rs2066714	Case (n = 110)	Control (n = 110)
A	87 (0.39)	203(0.92)
G	133(0.60)	17(0.07)
AA	5 (4.55%)	93 (84.55%)
AG	77 (70%)	17 (15.45%)
GG	28 (25.45%)	0
**rs757194699**	**Case** **(n = 150)**	**Control** **(n = 150)**
A	110(0.36)	236(0.78)
C	190(0.63)	64(0.21)
AA	0	86 (57%)
AC	110 (73%)	64 (43%)
CC	40 (27%)	0

**Table 2:** Association of rs757194699 and rs2066714 with diabetic dyslipidemia, N = 300, adjusted by sex + age + HDL).

SNP	Genotype	Cases	Controls	OR (95% CI)	*p*-Value	AIC	BIC
rs757194699 Overdominant	A/A-C/C	40 (26.7%)	86 (57.3%)	1.00	0.0011	166.6	185.1
	A/C	110 (73.3%)	64 (42.7%)	3.84 (1.67–8.82)			
*rs2066714* Codominant	A/A	5 (4.5%)	93 (84.5%)	1.00	<0.0001	75	95.3
	A/G	77 (70%)	17 (15.4%)	39.61 (9.97–157.32)			
	G/G	28 (25.4%)	0 (0%)	0.00 (0.00-NA)			
Dominant	A/A	5 (4.5%)	93 (84.5%)	1.00	<0.0001	77.9	94.8
	A/G-G/G	105 (95.5%)	17 (15.4%)	59.59 (15.19–233.81)			
Overdominant	A/A-G/G	33 (30%)	93 (84.5%)	1.00	<0.0001	110. 1	127
	A/G	77 (70%)	17 (15.4%)	9.75 (3.16–30.11)			
Log-additive	---	---	---	42.15 (11.08–160.40)	<0.0001	73.2	90.1

Variants in both the homozygous and heterozygous state were more common in males than females in the case and control groups. Table 3 shows that trend analysis revealed a significant association of rs2066714 in heterozygous with sex, but this was more significant in males (OR:45.42 (9.23–223.45)) than females (OR: 25.64 (1.75–376.06)).

### 2.3. Sanger Sequencing of Exon 5 of the ABCA1 Gene

**Figure 1:**
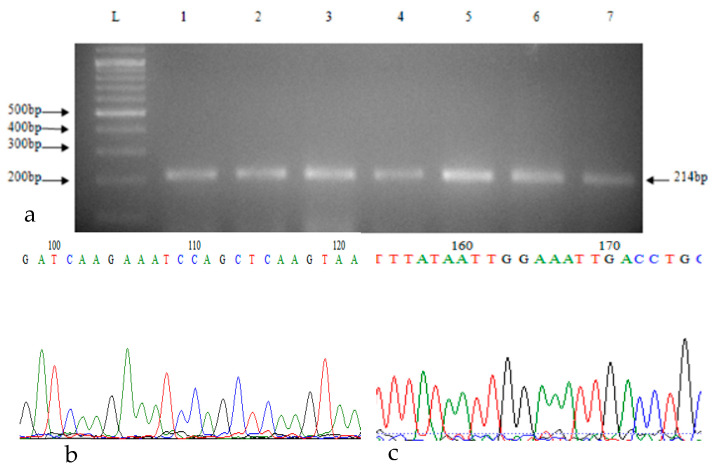
(**a**) PCR of exon 5 of *ABCA1* gene, (**b**,**c**) Sanger sequencing of *ABCA1* gene.

The 214 bp product was amplified at an annealing temperature of 60.1 °C (Figure 1a). Sanger sequencing demonstrated a normal pattern in the nucleotide sequences in exon 5 (Figure 1b,c) without any change between cases and controls. 

### 2.4. In Silico ABCA1 Docking with ApoA1

**Figure 2:**
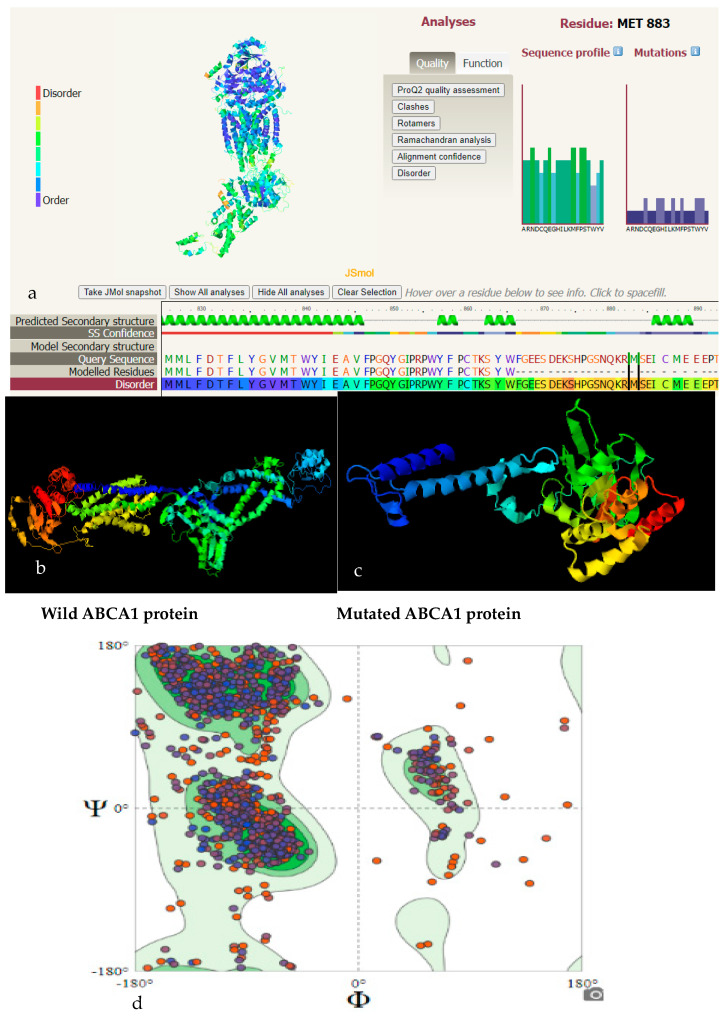
(**a**) Phyre2 investigator representing the disorder likelihood of I883M variant. (**b**) Wild Protein Model (C-score = −0.66 Model, Estimated TM-score = 0.63 ± 0.14, Estimated RMSD = 10.7 ± 4.6Å). (**c**) Mutant Protein Model designed with I-Tasser (C-score = 0.14 Model, Estimated TM-score = 0.73 ± 0.11, Estimated RMSD = 5.9 ± 3.7Å). (**d**) Ramachandran plot to assess protein structure quality.

The polymorphism rs2066714 A > G substituted isoleucine with methionine at position 883 (I883M). The polymorphism rs757194699 A > C substituted lysine with glutamine at position 1587 (K1587Q). These substitutions made changes to the structural features of the ABCA1 protein (Figure 2a–c), predicted by Swiss modelling and I-Tasser. The I883M substitution hence appeared to have a high disorder gain likelihood, and Mutation Taster predicted it to be a disease-causing polymorphism with possible protein feature changes. However, K1587Q appeared to have a low probability of disease-causing polymorphisms. PolyPhen2 represented the variant as benign, with a score of 0.292. Figure 2d shows the Ramachandran plot of the ABCA1 model chosen for docking; we determined that 90.86% of residues were in favored and allowed regions. The majority of secondary structure consist of right handed α helix, parallel and anti-parallel β sheets plates along with right twisted β sheets and left handed α helix. 

**Figure 3:**
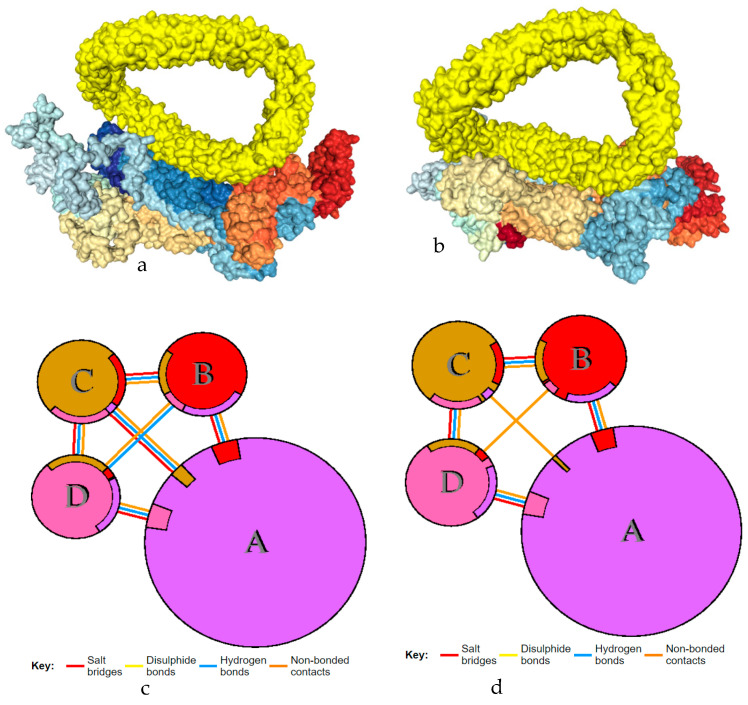
Protein docking of ABCA1 with apoA1 via HDOCK. (**a**) Wild l ABCA1 (Docking score −283.10, confidence score 0.9347, ligand rmsd (Å), 236.12). (**b**) I883M/K1587Q mutant ABCA1 protein (Docking score −291.80, confidence score, 0.9446, ligand rmsd (Å) 158.34. (**c**) Wild ABCA1 (A) binding interactions with three chains of apoA1. (**d**) Mutated ABCA1 (A) binding interactions with three chains of apoA1.

The two polymorphisms in combination has created changes in the 3D configuration of ABCA1 and binding interactions with apoA1 (Figure 3b) compared to the native ABCA1 protein (Figure 3a). A protein–protein interaction was identified, but clear change in the structure of ABCA1 was also noted. Figure 3c & d shows molecular binding sites at the ABCA1 and apoA1 protein–protein interface, generated using PDBsum. This showed the interaction of chains B, C, and D of apoA1 with ABCA1. This interaction was established through salt bridges, hydrogen bonds, and non-bonded contacts. These interactions were observed changed in mutated ABCA1-apoA1 complex (Table 4). The interacting residues of ABCA1 and chains B, C, and D of apoA1 are shown in Figures S1–S3 before and after the polymorphisms. 

## 3. Discussion

Diabetic dyslipidemia is a complex polygenic metabolic disorder; multiple factors contribute to its development. The *ABCA1* gene encodes a transporter protein that plays a role in cholesterol homeostasis and the synthesis of mature HDL-C particles. Genetic variations in the *ABCA1* gene alter the cholesterol transport mechanism, impacting lipid metabolism. Various genome-wide association studies have established a link between disease susceptibility and polymorphisms within the genome. The present study focused on the variations in the *ABCA1* gene in association with diabetic dyslipidemia, as Pakistan includes a diverse range of ethnic groups. 

We selected the rs2066714 (I883M) and rs757194699 (K1587Q) polymorphisms located in the exonic region of the *ABCA1* gene to test their possible roles in diabetic dyslipidemia. We observed the homozygous GG genotype of rs2066714 (I883M) only in the case group, while the AG genotype was significantly more frequent in the case group compared to the control group. We observed more deranged levels of HDL, LDL, and TG in individuals who were carriers of the AG genotype than in GG genotypes. However, a direct correlation of the studied polymorphism with lipid parameters was not found. Corresponding to our findings, previous studies documented the association of the genotypes of the I883M variant with lipid level difference in obese children and adolescents, and hyperlipidemic population [25,26]. Our genetic model findings suggest that the polymorphism rs2066714 (I883M) implies a likelihood of disease onset in the future in a population carrying the GG or AG genotype. Our study presents a significant association for this SNP (*p*-value < 0.001) in all genetic models.

Genetic anomalies in the *ABCA1* gene may play a role in reducing HDL-C levels. One author discovered a novel variation in the *ABCA1* gene, exon 14, in individuals with low HDL-C [27]. In addition, previous studies have reported a correlation between *ABCA1* polymorphisms and the onset of diabetes and its complications [23,28,29]. Wang et al. demonstrated a significant association of variant I883M in genetic models (AA vs. GG = *p*-value 0.010 and recessive *p*-value 0.011) with coronary heart disease [30]. Lu et al. investigated *ABCA1* gene variants regarding susceptibility to coronary artery disease and detected the G allele of the variant as a risk allele (*p*-value < 0.05) for disease susceptibility. The subjects bearing GA genotype were observed to have high TC and low LDL levels. However, the gene polymorphism and disease severity (CAD) did not show any statistically significant association [31].

We found the homozygous GG genotype in 25.45% of subjects, with it being present only in diseased subjects, while Kolovo et al. revealed only 1.9% of the MM genotype in their study and did not find any correlation between the gene variant and lipid variables [32]. In the I883M variant, the replacement of isoleucine with methionine does not mark a change in the hydrophobicity of amino acid but both are different in chemical nature and solubility. Methionine contains sulphur in side chain while isoleucine is an aliphatic amino acid. It does change the electrostatic interactions and changes the binding pocket of ABCA1 and apoA1. Moreover, SNP analysis performed in Phyre2 investigations has revealed the position of an amino acid with high disorder gain. Mutation Taster presents the variant as a disease-causing polymorphism. 

Our study reports the genotyping of rs757194699 (K1587Q) in diabetic dyslipidemia patients for the first time in Pakistan. The higher frequency of CC and AC genotypes in the cases marks likely susceptibility to diabetic dyslipidemia disease later in life. In K1587Q, the amino acid changes from being basic to non-polar, which confers some structural change to the protein. These suggested alterations in the binding pocket of ABCA1 with apoA1, produce defects in molecular function and reverse cholesterol transport. 

The molecular docking of mutated ABCA1 carrying both polymorphisms has created remarkable distortion in the 3D structure and binding pocket with apoA1. We have created a model of a mutated ABCA1 protein. As shown in Table 4, the binding sites and interactions were changed between mutated ABCA1 and apoA1. The apoA1 protein is a homo-tetramer, with the chains A, B, C, and D. Mirza et al. [33] have reported anti-atherogenic properties in fucoidan. They found interactions between APOA1 and the ECD1 domain of ABCA1, thus stimulating cholesterol efflux via the increased activity of fucoidan. However, we used all four chains of apoA1 for docking with the ABCA1 protein. Clear changes in binding sites were identified between ABCA1 and apoA1. These protein–protein docking results suggest changes in the function of apoA1 and ABCA1 because both are involved in lipid and cholesterol transport. Moreover, we speculate the change in the binding of cholesterol with ABCA1 and apoA1 proteins due to the presence of these polymorphisms. Recently, the role of ABC transporters in the physiology of the brain has been documented in Alzheimer’s. Their role was identified in glial cells, where several ABC transporters were engaged in cellular brain homeostasis. They also elucidated the functions of ABC transporters in *Huntington’s disease* and *experimental autoimmune encephalomyelitis* [34]. 

Polymorphisms play critical roles in genetic susceptibility, and we are currently targeting them to gain better insights of the genetic aspects of diseases. This is the first study conducted on our population to determine genetic susceptibility for diabetic dyslipidemia. Diabetic dyslipidemia is a major risk factor in the onset of cardiovascular diseases. Our data provide the insight that these polymorphisms represent a contributing factor in the development of diabetic dyslipidemia. This is the first study to dissect the molecular architecture of exon 5 of the *ABCA1* gene for our type 2 diabetic population with dyslipidemia. The previous data do not report any genetic screening of the studied gene exon regarding diabetic dyslipidemia. However, we have not detected any variation in the studied exon, and disease susceptibility may be attributed to other exons of the gene.

Genetic factors are considered significant contributors to any disease onset, and there is a pressing need to elucidate their contribution to personalized medicine. Genetic studies open up new horizons in terms of diagnosis, prognosis, and therapeutic strategies based on a personalized approach. ABCA1 is a key lipid-regulatory protein and is involved in maintaining HDL metabolism. Variations in the gene disrupt the function of the protein and can alter the normal mechanism. *ABCA1* gene variants have been observed to be positively associated with abnormal lipid profiles among diabetics. It is crucial to uncover the role of molecular determinants to explore their mechanism and develop therapeutic modalities. The data pool generated from genetic association studies represents a new step along the therapeutic path. This can be further integrated into personalized medicine or a particular population-based therapeutic target. 

### Study Strengths and Limitations

This study has strengths, as well as some limitations. This study aimed to pinpoint genetic susceptibility to disease onset by selecting newly diagnosed type II diabetic patients with dyslipidemia first time. This study first time has generated genetic data of SNPs in *ABCA1* gene in our newly diagnosed type II diabetic patients with dyslipidemia. The findings show the genetic modulation of diabetic dyslipidemia, which could be used to devise better therapeutic strategies in future. 

We were not able to enroll large samples due to limited participation and results are restricted to studied subjects. We did not take into account environmental contributors due to limited resources. The study was conducted on a diverse group of individuals. However, our study was not ethnicity-based, and large samples need to be included in order to obtain comprehensive data on the studied polymorphism. 

## 4. Materials and Methods

### 4.1. Study Design and Sample Collection

This case–control study was performed on 330 subjects, following formal approval from the institutional ethical review committee of the Army Medical College and conducted in compliance with the principles of the Declaration of Helsinki [35], with written consent from each participant. Patients newly diagnosed with type 2 diabetic dyslipidemia (cases) and healthy individuals (control) were recruited from Pak Emirates Military Hospital, Rawalpindi. A non-probability purposive sampling technique was used. Subjects with gestational diabetes, hypertension, type 1 diabetes, or comorbidities, along with those on any lipid-lowering therapy, were subject to exclusion from the study. The comorbidities considered subject to the exclusion criteria in consultation with the medical specialist were renal, lung, and cardiac diseases, and cancer. Out of 330, 150 cases and 150 controls were screened for the rs757194699 (K1587Q) gene variant, 110 cases and 110 controls were screened for the rs2066714 (I883M) polymorphism, and 90 cases underwent Sanger sequencing. rs2066714 (I883M) was selected based on the literature cited, due to its association with lipid levels and CHD, while rs757194699 (K1587Q) was taken as a new pathogenic variant from dbSNP database. 

### 4.2. Molecular Genotyping of the ABCA1 Gene Variants rs757194699 (K1587Q) and rs2066714 (I883M)

Venous blood samples were collected in EDTA tubes from all the subjects, with stringent hygienic measures taken. The phenol–chloroform method [36] was used to extract genomic DNA. Polymerase chain reaction (PCR) was performed using primers 5′-gagaagagccaccctggttccaaccagaagaggat-3′ and 5′-agaaaggcaggagacatcgctt-3′, which were described in the study [26] for the genotyping of the variant *rs2066714 A > G* (I883M). The 132 bp PCR products were incubated with *Eco32I* enzyme (isoschizomer of *EcoRV*, Thermo Fisher Scientific, Waltham, MA, USA) at 37 °C for 2 h, which produced 132 bp fragment for mutant G allele and 97 and 35 bp for the A allele. 

Allele-specific polymerase chain reaction was employed for genotyping of *rs757194699 A > C* (K1587Q). The allele-specific primers were designed through a Web-based allele-specific primer design tool (WASP) available at (https://bioinfo.biotec.or.th/WASP, accessed on 3 February 2022). Primer sequence was as follows: wild forward 5′-agatttatgacaggactggacacca-3′, mutant forward 5′-agatttatgacaggactggacaccc-3′, and common reverse 5′-tgccaactttaccatgagtt-3′. The PCR was performed as initial denaturation at 95 °C for 5 min, followed by 35 cycles of denaturation at 95 °C for 30 s, annealing at 61.5 °C for 30 s, extension at 72 °C for 30 s, and final extension at 72 °C for 7 min. Two separate reactions were performed for the wild-type and mutant alleles. The PCR produced a product size of 128 bp resolved on 2% agarose gel to determine the mutant and wild-type alleles. 

### 4.3. Genetic Screening of Exon 5 of ABCA1 

For the genetic screening of exon 5 of the *ABCA1* gene, primers were designed on online bioinformatics software tool “Primer 3Plus”, available at (https://www.bioinformatics.nl/cgi-bin/primer3plus, accessed on 5 July 2021), with the forward primer being 5′-gcctttcgccttttcttgca-3′ and the reverse primer being 5′actctctttccctggtgcag-3′. The reaction was prepared using 50 ng genomic DNA in a total reaction volume of 25 μL containing 1x*Taq* buffer, 1.5 mM MgCl_2_, 0.2 mM dNTPs, 0.5 units *Taq* DNA polymerase (Thermo Fisher Scientific, Waltham, MA, USA), nuclease free water and 1 pmol/μL of each primer (Macrogen Inc., Seoul, South Korea). The PCR program was set as follow: initial denaturation at 95 °C for 3 min, denaturation at 95 °C for 30 s, annealing at 60.1 °C for 35 s, and extension at 72 °C for 30 s, followed by 35 repeats in a thermocycler (Bio-Rad T100TM Inc., Hercules, CA, USA). The 214 bp desired gene fragment was obtained and purified by using GeneJET PCR Purification-Kit (Thermo Fisher Scientific, Waltham, MA, USA). The sequencing reactions were performed using dideoxy terminator cycle sequencing (GenomeLab-DTCS) kit (Beckman Coulter, Fullerton, CA, USA) on an automated DNA Sequencer (CEQ8000, Beckman Coulter, Fullerton, CA, USA). The *ABCA1* gene sequence variation designation was based on the NCBI genomic reference sequence NG_007981.1 (corresponding to Ensembl gene ENSG00000165029). The *Human Genome Variation Society nomenclature* (version 15.11) (http://varnomen.hgvs.org/, accessed on 5 July 2021) was employed to label gene variants. The obtained DNA sequence data were analyzed for the detection of nucleotide sequence variation through BioEdit version 7.2.5 software, global sequence alignment on Basic Local Alignment Search Tool (BLAST), and searching online genome databases at 1000 Genome, gnomAD. 

### 4.4. In Silico ABCA1 Docking with ApoA1

I-Tasser [37], Swiss Model [38], and Phyre2 [39] online bioinformatic suites were used to build the models of ABCA1 protein with alternate amino acids in the sequence for the variants I883M and K1587Q. ClusPro [40] and HDOCK [41] were employed to assess the protein docking of apoA1 with ABCA1 protein using models built with the Swiss Model. They were further visualized on PyMol (PyMol v.2.5.)

Genetic variants were analyzed using Polyphen2 (http://genetics.bwh.harvard.edu/pph2/ accessed on 13 October 2023) and Mutation Taster (https://www.mutationtaster.org/ accessed on 13 October 2023) algorithms. Polyphen2 was used to predict the possible effect of amino acid substitution in our observed variants for the likelihood damage to the structure of protein. 

### 4.5. Statistical Data

The data of both genetic variants were statistically analyzed on statistical software SNPStats [42] available at (https://www.snpstats.net/start.htm, accessed on 20 November 2023). Hardy–Weinberg Equilibrium (HWE), allelic and genotypic frequency and associations were tested using Fisher’s exact test, Chi-square (χ^2^) test. Logistic regression analysis was applied to calculate the odds ratios (ORs) with 95% confidence intervals (95% CIs) for the association of I883M and K1587Q with the risk of diabetic dyslipidemia. In the presence of diverse data on different population regarding these polymorphisms, all four genetic models were used. A *p*-value less than or equal to 0.05 was considered statistically significant. 

## 5. Conclusions

Genetic studies help our understanding of the molecular pathophysiology of diseases. ABCA1 is a key lipid-regulating protein in cholesterol efflux and HDL metabolism. ABCA1 gene variations have been considered as an early diagnosing strategy to investigate disease susceptibility. We conclude that rs2066714 and rs757194699 are significantly associated with susceptibility to diabetic dyslipidemia. The G allele of rs2066714 and the C allele of rs757194699 in the *ABCA1* gene are found to be risk alleles in the development of dyslipidemia in type 2 diabetes. These polymorphisms predominantly altered the binding site of ABCA1 with apoA1 to disturb the cholesterol transport pathway. These genetic variants could possibly be used as potential biomarkers in the near future.

## Figures and Tables

**Table 3:** Interaction analysis of rs2066714 with the covariate of sex (adjusted by AGE+HDL).

Sex	Genotypes	Status	OR (95% CI)	
Female		Control	Case	
	I/I	33	1	1.00
	I/M	6	14	25.64 (1.75–376.06)
	M/M	0	8	---
Male	I/I	60	4	1.00
	I/M	11	63	45.42 (9.23–223.45)
	M/M	0	20	---

Test for interaction in the trend: 0.94.

**Table 4:** Summary of docking interface statistics for protein–protein interactions between ABCA1 and apoA1.

**Interface statistics for wild ABCA1 and apoA1 interactions**						
**Chains**	**No. of** **interface** **residues**	**Interface** **area (Å^2^)**	**No. of** **salt** **bridges**	**No. of** **disulphide** **bonds**	**No. of** **hydrogen** **bonds**	**No. of** **non-bonded** **contacts**
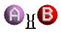	82:84	4659:4718	25	-	50	515
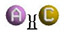	49:38	2505:2651	6	-	7	237
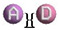	72:71	4502:4534	6	-	28	348
**Interface statistics for mutated ABCA1 and apoA1**						
**Chains**	**No. of** **interface** **residues**	**Interface** **area (Å^2^)**	**No. of** **salt** **bridges**	**No. of** **disulphide** **bonds**	**No. of** **hydrogen** **bonds**	**No. of** **non-bonded** **contacts**
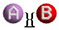	56:58	4034:4032	12	-	12	340
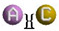	15:15	852:851	-	-	-	38
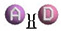	69:57	4287:4459	4	-	7	307

## Data Availability

The data is contained within the article and Supplementary Materials.

## Supplementary Materials

The following supporting information can be downloaded at: https://www.mdpi.com/article/10.3390/ijms25126796/s1.
