# Supplementary material for: Identification and Molecular Simulation of Genetic Variants in ABCA1 Gene Associated with Susceptibility to Dyslipidemia in Type 2 Diabetes
Source: Int J Mol Sci. 2024 Jun 20;25(12):6796. doi: 10.3390/ijms25126796 (PMC11203815; doi:10.3390/ijms25126796)
Supplement: Supplementary file 1 [file ijms-25-06796-s001.zip › Figure S3.pdf]

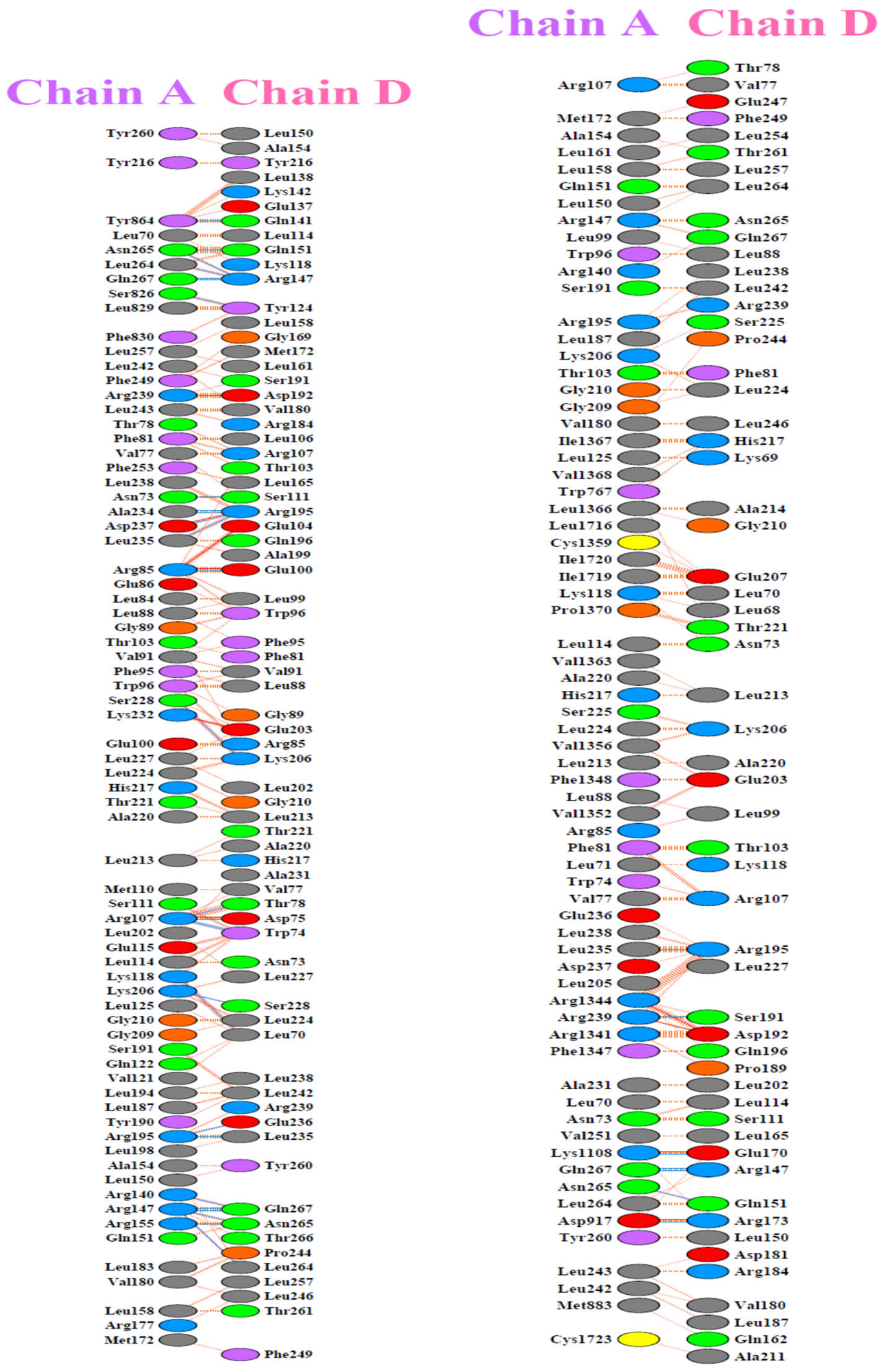

A

B

Figure S3. Schematic diagram of interaction of ABCA1 and APOA1. Each circle represents residues of each protein chain while colored lines represent interactions. A) wild ABCA1 with D chain of APOA1. B) Mutated ABCA1 with D chain of APOA1.
